# Correction

**DOI:** 10.1093/biostatistics/kxae029

**Published:** 2024-08-26

- Biostatistics, Volume 25, Issue 3, July 2024, Pages 599–616, https://doi.org/10.1093/biostatistics/kxad015
- Biostatistics, Volume 25, Issue 3, July 2024, Pages 769–785, https://doi.org/10.1093/biostatistics/kxad020
- Biostatistics, Volume 25, Issue 3, July 2024, Pages 702–717, https://doi.org/10.1093/biostatistics/kxad021
- Biostatistics, Volume 25, Issue 3, July 2024, Pages 633–647, https://doi.org/10.1093/biostatistics/kxad022
- Biostatistics, Volume 25, Issue 3, July 2024, Pages 852–866, https://doi.org/10.1093/biostatistics/kxad023
- Biostatistics, Volume 25, Issue 3, July 2024, Pages 617–632, https://doi.org/10.1093/biostatistics/kxad024
- Biostatistics, Volume 25, Issue 3, July 2024, Pages 786–800, https://doi.org/10.1093/biostatistics/kxad025
- Biostatistics, Volume 25, Issue 3, July 2024, Pages 885–903, https://doi.org/10.1093/biostatistics/kxad026
- Biostatistics, Volume 25, Issue 3, July 2024, Pages 904–918, https://doi.org/10.1093/biostatistics/kxad027
- Biostatistics, Volume 25, Issue 3, July 2024, Pages 754–768, https://doi.org/10.1093/biostatistics/kxad028
- Biostatistics, Volume 25, Issue 3, July 2024, Pages 867–884, https://doi.org/10.1093/biostatistics/kxad030
- Biostatistics, kxad031, https://doi.org/10.1093/biostatistics/kxad031
- Biostatistics, Volume 25, Issue 3, July 2024, Pages 736–753, https://doi.org/10.1093/biostatistics/kxad032
- Biostatistics, kxad033, https://doi.org/10.1093/biostatistics/kxad033
- Biostatistics, Volume 25, Issue 3, July 2024, Pages 666–680, https://doi.org/10.1093/biostatistics/kxad034
- Biostatistics, kxad035, https://doi.org/10.1093/biostatistics/kxad035
- Biostatistics, Volume 25, Issue 3, July 2024, Pages 833–851, https://doi.org/10.1093/biostatistics/kxad036
- Biostatistics, Volume 25, Issue 3, July 2024, Pages 818–832, https://doi.org/10.1093/biostatistics/kxad037
- Biostatistics, Volume 25, Issue 3, July 2024, Pages 919–932, https://doi.org/10.1093/biostatistics/kxad038
- Biostatistics, kxae001, https://doi.org/10.1093/biostatistics/kxae001
- Biostatistics, kxae002, https://doi.org/10.1093/biostatistics/kxae002
- Biostatistics, kxae003, https://doi.org/10.1093/biostatistics/kxae003
- Biostatistics, kxae005, https://doi.org/10.1093/biostatistics/kxae005
- Biostatistics, kxae006, https://doi.org/10.1093/biostatistics/kxae006
- Biostatistics, kxae007, https://doi.org/10.1093/biostatistics/kxae007
- Biostatistics, kxae008, https://doi.org/10.1093/biostatistics/kxae008
- Biostatistics, kxae009, https://doi.org/10.1093/biostatistics/kxae009
- Biostatistics, kxae011, https://doi.org/10.1093/biostatistics/kxae011
- Biostatistics, kxae012, https://doi.org/10.1093/biostatistics/kxae012
- Biostatistics, kxae013, https://doi.org/10.1093/biostatistics/kxae013
- Biostatistics, kxae014, https://doi.org/10.1093/biostatistics/kxae014
- Biostatistics, kxae015, https://doi.org/10.1093/biostatistics/kxae015
- Biostatistics, kxae016, https://doi.org/10.1093/biostatistics/kxae016

The originally published PDF versions of the manuscripts below contained incorrect links to their supplementary material. The erroneous link has been corrected within each of the following articles.

- https://doi.org/10.1093/biostatistics/kxad015
- https://doi.org/10.1093/biostatistics/kxad020
- https://doi.org/10.1093/biostatistics/kxad021
- https://doi.org/10.1093/biostatistics/kxad022
- https://doi.org/10.1093/biostatistics/kxad023
- https://doi.org/10.1093/biostatistics/kxad024
- https://doi.org/10.1093/biostatistics/kxad025
- https://doi.org/10.1093/biostatistics/kxad026
- https://doi.org/10.1093/biostatistics/kxad027
- https://doi.org/10.1093/biostatistics/kxad028
- https://doi.org/10.1093/biostatistics/kxad030
- https://doi.org/10.1093/biostatistics/kxad031
- https://doi.org/10.1093/biostatistics/kxad032
- https://doi.org/10.1093/biostatistics/kxad033
- https://doi.org/10.1093/biostatistics/kxad034
- https://doi.org/10.1093/biostatistics/kxad035
- https://doi.org/10.1093/biostatistics/kxad036
- https://doi.org/10.1093/biostatistics/kxad037
- https://doi.org/10.1093/biostatistics/kxad038
- https://doi.org/10.1093/biostatistics/kxae001
- https://doi.org/10.1093/biostatistics/kxae002
- https://doi.org/10.1093/biostatistics/kxae003
- https://doi.org/10.1093/biostatistics/kxae005
- https://doi.org/10.1093/biostatistics/kxae006
- https://doi.org/10.1093/biostatistics/kxae007
- https://doi.org/10.1093/biostatistics/kxae008
- https://doi.org/10.1093/biostatistics/kxae009
- https://doi.org/10.1093/biostatistics/kxae011
- https://doi.org/10.1093/biostatistics/kxae012
- https://doi.org/10.1093/biostatistics/kxae013
- https://doi.org/10.1093/biostatistics/kxae014
- https://doi.org/10.1093/biostatistics/kxae015
- https://doi.org/10.1093/biostatistics/kxae016

